# The duration of diarrhea and fever is associated with growth faltering in rural Malawian children aged 6-18 months

**DOI:** 10.1186/1475-2891-10-25

**Published:** 2011-03-20

**Authors:** Ariana Weisz, Gus Meuli, Chrissie Thakwalakwa, Indi Trehan, Kenneth Maleta, Mark Manary

**Affiliations:** 1 Washington University in St. Louis, One Children's Place, Campus Box 8116, St. Louis, MO, 63110, USA; 2 University of Malawi College of Medicine, Private Bag 360, Blantyre 3, Malawi; 3 USDA Children's Nutrition Research Center, Baylor College of Medicine, 1100 Bates St., Houston, TX, 77030, USA

## Abstract

Nutrition support programs that only focus upon better complementary feeding remain an insufficient means of limiting growth faltering in vulnerable populations of children. To determine if symptoms of acute infections correlate with the incidence of growth faltering in rural Malawian children, the associations between fever, diarrhea, and cough with anthropometric measures of stunting, wasting, and underweight were investigated. Data were analyzed from a trial where 209 children were provided with adequate complementary food and followed fortnightly from 6-18 months of age. Linear mixed model analysis was used to test for associations. Diarrheal disease was inversely associated with changes in height-for-age Z-score (HAZ), mid-upper arm circumference Z-score (MUACZ), and weight-for-age Z-score (WAZ). Fever was also inversely associated with changes in MUACZ and WAZ. These results suggest that initiatives to reduce febrile and diarrheal diseases are needed in conjunction with improved complementary feeding to limit growth faltering in rural Malawi.

## Findings

While the proximate causes of death among children worldwide are predominantly infectious in nature, undernutrition remains an underlying factor in approximately half of all deaths among children less than five years of age [1]. Efforts to address childhood undernutrition as a means to decrease overall childhood mortality are therefore appropriately prominent on the global health agenda. At the same time, the contributions of acute childhood infections to growth faltering, with its associated catabolic metabolism, have long been recognized [2,3]. For example, diarrheal disease may be responsible for up to 80% of childhood growth retardation [3]. This infection-malnutrition link has so far been largely unexplored in Malawi.

Wasting, defined as a mid-upper arm circumference Z score (MUACZ) or a weight-for-height Z score (WHZ) less than -2, affects 4%-6% of Malawian children under five [4,5]. Stunting, defined as a height-for-age Z score (HAZ) less than -2, affects 48%-53% of Malawian children under five [6,7]. As in other populations [8], weight faltering begins at about 6 months of age for rural Malawian children and is frequently attributed to poor complementary feeding [8,9].

Data collected during a prospective longitudinal clinical effectiveness trial of complementary feeding in rural Malawian children aged 6-18 months [10] were analyzed to determine the relationship between symptoms of an acute infectious illness and growth. The primary study outcomes were the association of changes in markers of stunting (HAZ), wasting (MUACZ), and underweight (weight-for-age Z-score, WAZ) with mothers' reports of fever, cough, and diarrhea. Consent was obtained from all caretakers and the study was approved by the College of Medicine Research Ethics Committee at the University of Malawi and the Human Research Protection Office at Washington University in St. Louis.

Participants came from the families of subsistence farmers who have difficulty growing crops in the sandy soil surrounding nearby Lake Chilwa. The participants' homes were constructed of mud and thatch; electricity was not available to any residents. Mothers were the principal caretakers, often carrying the children on their backs throughout the day, and sleeping with the children at night. The habitual diet was monotonous: maize was the staple food, which was supplemented with small fish.

All children were breast fed *ad lib*, and in addition each was randomly assigned to receive isoenergetic amounts of one of two complementary foods, either a corn porridge fortified with fish powder or a micronutrient fortified peanut/soy spread. Both foods provided adequate amounts of macro- and micronutrients according to the World Health Organization's daily-recommended intakes [10,11].

Children were followed fortnightly for symptoms of infection by maternal report of fever, cough, or diarrhea in a structured interview conducted by village health workers resident in the child's own village. Fever was defined as mother's perception of increased body temperature and diarrhea as at least 3 loose stools per day. In preparation for the interview, mothers were alerted that this information would be solicited, offered calendars on which they could record their child's symptoms, and informed that no specific treatments would be offered by the study team on the basis of their responses. The child's weight and mid-upper arm circumference (MUAC) were measured monthly and length was measured every 3 months until the child reached 18 months of age. A socioeconomic score was determined by 3 characteristics: if the child's family lived in a home with a metal roof, owned a bicycle, or owned a radio, with 1 point given for each criterion met. Comparable proxies for socioeconomic status have been used previously in similar studies [12-14].

All children who were followed for at least 280 days were included in the data analyses. HAZ, WAZ, WHZ, and MUACZ were calculated using WHO Anthro 3.1 (WHO, Geneva) based on the WHO 2006 growth standards [15]. MUACZ was chosen as the measure of wasting instead of WHZ because MUAC was measured every month and thus could be more closely correlated with short term growth rates. Linear mixed model analyses were performed using PASW Statistics version 17 (2008, IBM, Somers, NY) to determine the effects of reported fever, cough, and diarrhea on changes in HAZ, WAZ, and MUACZ. Fixed effects included in the model were gender, complementary food type, access to clean water, enrollment WHZ, enrollment HAZ, enrollment MUAC, enrollment WAZ, number of siblings, and socioeconomic score. Model coefficients that did not include 0 in their 95% confidence interval were considered statistically significant.

A total of 209 children were included in the study (Table 1). The number of days of diarrhea reported was higher among children 6-12 months old than among children 12-18 months old (15 days vs. 7 days, p < 0.001, Table 2). HAZ decreased by approximately 0.5 Z scores during the 12 month observation period. A greater duration of diarrhea was associated with greater reductions in HAZ; greater duration of fever and diarrhea were associated with greater reductions in MUACZ; and greater duration of fever, diarrhea and cough were associated with greater reductions in WAZ during the observation period (Table 3). Gender, socioeconomic score, the type of supplemental food received, and access to clean water were not significantly associated with changes in HAZ, WAZ, or MUACZ.

**Table 1:** Demographic and anthropomorphic characteristics of study children (N = 209) at enrollment.

Characteristic	Mean ± SD or N (%)
Age, *mo*	6.3 ± 0.3
Male	95 (45%)
Number of siblings	2.1 ± 1.7
WAZ	-0.7 ± 1.1
WHZ	0.0 ± 1.0
HAZ	-1.0 ± 1.1
MUAC, *cm*	14.2 ± 1.2
MUACZ	0.1 ± 1.0
Slept under a bed net the previous night	92 (44%)
Received water from a clean source (deep borehole)	137 (66%)
Home with metal roof	8 (4%)
Family owns bicycle	145 (69%)
Family owns radio	140 (67%)

**Table 2:** Outcomes of study children at 6 to 12 months and 12-18 months (mean ± SD)

Outcome	6-12 mo	12-18 mo
WAZ change during interval	-0.4 ± 0.5	0.1 ± 0.4*
HAZ change during interval	-0.3 ± 0.6	-0.2 ± 0.5
MUACZ change during interval	-0.1 ± 0.8	0.0 ± 0.6
Days of fever during interval	11 ± 8.7	10 ± 8.8
Days of cough during interval	9 ± 7.9	12 ± 8.5*
Days of diarrhea during interval	15 ± 10.4	7 ± 8.2*

*p < 0.001 by two tailed paired t-test, comparing 6-12 mo interval with 12-18 mo interval

**Table 3:** Coefficients in linear mixed model analyses (95% CI).

Parameters	Change in HAZ	Change in MUACZ	Change in WAZ
*Categorical*			
Male gender	-0.030 (-0.107, 0.047)	0.023 (-0.025, 0.072)	-0.013 (-0.035, 0.008)
Received water from a clean source (deep borehole)	-0.016 (-0.095, 0.063)	-0.003 (-0.047, 0.042)	-0.002 (-0.023, 0.020)
Received fortified peanut/soy spread	0.010 (-0.068, 0.088)	0.013 (-0.031, 0.057)	0.009 (-0.012, 0.031)
*Continuous*			
Socioeconomic score	-0.004 (-0.052, 0.043)	-0.005 (-0.031, 0.022)	-0.0009 (-0.014, 0.012)
Days of fever	-0.004 (-0.011, 0.002)	-0.005 (-0.007, -0.003)	-0.003 (-0.005, -0.002)
Days of cough	0.004 (-0.002, 0.010)	-0.0008 (-0.003, 0.002)	-0.001 (-0.002, -0.0001)
Days of diarrhea	-0.007 (-0.012, -0.002)	-0.005 (-0.007, -0.003)	-0.005 (-0.006, -0.0004)
Enrollment WHZ	0.041 (0.005, 0.077)	0.008 (-0.024, 0.040)	-0.021 (-0.031, -0.011)
Enrollment HAZ	-0.058 (-0.094, -0.023)	0.013 (-0.011, 0.037)	-0.006 (-0.016, 0.004)
Enrollment MUAC	---	-0.034 (-0.068, -0.001)	---

The primary findings of this study are that among rural Malawian children 6 to 18 months of age, more days of diarrhea was associated with greater decreases in HAZ, MUACZ, and WAZ and that more days of fever was associated with greater decreases in MUACZ and WAZ. More days of cough was also found to be inversely associated with change in WAZ.

These findings relating diarrhea to growth faltering may be related to nutrient malabsorption during and following the diarrheal episode. The growth faltering observed with respiratory and febrile illnesses may be related to the increased metabolic demand and decreased food intake that is common during such illnesses. HIV status was not routinely checked for the children in this study.

Our results are consistent with other African studies investigating the incidence of infectious morbidity and growth faltering. In a Ghanaian study of children aged 6-12 months, the prevalence of diarrhea was inversely associated with growth in both weight and length [16]. In a carefully conducted Gambian study in children 0-24 months, an increased prevalence of diarrhea and malaria were associated with growth faltering [17]. In northern Sudan, an inverse association between diarrheal and respiratory symptoms and gains in weight and height was found in a large prospective study of children aged 6-72 months [18]. In rural Uganda, a large cross-sectional survey of children aged 0-23 months found that a history of fever was associated with wasting and stunting [19]. Given the variation in prevalence of different infections in other settings, our findings should be extrapolated with caution to other populations, especially those outside rural sub-Saharan Africa.

The primary limitation of the study lies in the uncertainty inherent in the fortnightly parental recall method for fever, cough, and diarrhea. More frequent collection of this information may have resulted in more accurate estimates of the incidence and duration of acute infections. Other studies in Africa have collected such morbidity information in a similar manner at intervals that vary from 7 to 28 days [16-19], whereas some studies in Asia and South America have assessed this as often as daily [20,21]. Although not ideal, this fortnightly surveying interval is likely adequate to make reasonable clinical estimates of these infectious symptoms [22]. We can assume that among this study population receiving complementary food that our monitoring detected all cases of serious illness and provided a fair estimate of less severe morbidity.

It is remarkable that even though these breastfed children were all enrolled in a feeding study which provided adequate amounts of complementary food, growth faltering still occurred. This observation emphasizes that measures to control infectious morbidity must accompany nutritional interventions in order to optimize growth during this critical period. Widespread introduction of new rotavirus vaccines [23] and the continued development of safe water supplies [24] are likely to provide the greatest decrease in diarrheal morbidity on a population level. Nevertheless, small-scale interventions such as education about proper handwashing and the distribution of soap have proven quite effective as well [25] - interventions that could be feasibly integrated into a supplemental feeding program aimed at limiting growth faltering in this age group. As emphasized by the Integrated Management of Childhood Illness strategy [26], a synergy of approaches, both nutritional and anti-infective, should be pursued and tested in operational settings, as significant growth faltering was still observed during this strictly nutritional intervention.

## Competing interests

The authors declare that they have no competing interests.

## Authors' contributions

MM and KM designed and conceived the study, MM, AW, GM, CT, IT acquired, analyzed and interpreted the data, all authors helped to draft and finalize the manuscript. All authors have seen the final copy of the manuscript and agree with its contents.
